# Synchronous intrathyroidal parathyroid carcinoma and thyroid carcinoma: case report and review of the literature

**DOI:** 10.1186/s12902-021-00724-7

**Published:** 2021-04-07

**Authors:** Nadia De Falco, Giuseppe Santangelo, Fabrizio Chirico, Angelo Cangiano, Maria Giulia Sommella, Angelo Cosenza, Andrea Ronchi, Marina Accardo, Gianluca Pellino, Domenico Parmeggiani, Silvestro Canonico, Massimo De Falco

**Affiliations:** 1 General Surgery Unit, Department of Advanced Medical and Surgical Sciences, University of Campania “Luigi Vanvitelli”, Piazza Miraglia, 80138 Naples, Italy; 2 Maxillofacial Surgery Unit, Federico II University, Naples, Italy; 3 Division of Morphopathology, Department of Advanced Medical and Surgical Sciences, University of Campania “Luigi Vanvitelli”, Naples, Italy

**Keywords:** Thyroid cancer, Parathyroid cancer, Hyperparathyroidism, Intrathyroidal parathyroid, Surgical approach

## Abstract

**Background:**

Parathyroid carcinoma is a rare endocrine malignancy, rarer when synchronous with a non medullary well differentiated thyroid carcinoma. Parathyroid carcinoma accounts of 0.005% of all malignant tumors and it is responsible for less than 1% of primary hyperparathyroidism. The intrathyroidal localization of a parathyroid gland is not frequent with a reported prevalence of 0.2%. Carcinoma of parathyroids with intrathyroidal localization represents an even rarer finding, reported in only 16 cases described in literature. The rare constellation of synchronous parathyroid and thyroid carcinomas has prompted us to report our experience and perform literature review.

**Case presentation:**

We herein report a case of a 63-years-old man with multinodular goiter and biochemical diagnosis of hyperparathyroidism. Total thyroidectomy with radio-guide technique using gamma probe after intraoperative sesta-MIBI administration and intraoperative PTH level was performed. The high radiation levels in the posterior thyroid lobe discovered an intrathyroidal parathyroid. Microscopic examination revealed a parathyroid main cell carcinoma at the posterior thyroidal left basal lobe, a classic papillary carcinoma at the same lobe and follicular variant of papillary carcinoma at the thyroidal right lobe. To the best of our knowledge, this is the first case documenting a synchronous multicentric non medullary thyroid carcinomas and intrathyroidal parathyroid carcinoma.

**Conclusions:**

Our experience was reported and literature review underlining challenging difficulties in diagnostic workup and surgical management was carried out.

## Background

The intrathyroidal localization of a parathyroid gland is not frequent, with a reported prevalence of 0.2% [1]. Intrathyroidal parathyroids (IP) could also become pathological and primary hyperparathyroidism supported by an IP has an incidence of 1% [2].

Parathyroid carcinoma (PC) accounts of 0.005% of all malignant tumors and it is responsible for less than 1% of primary hyperparathyroidism [3, 4]. Carcinoma of parathyroids with intrathyroidal localization represents an even rarer finding, reported in only 16 cases described in literature [5–20].

The association between hyperparathyroidism and non-medullary well differentiated thyroid carcinoma is found in 2.4–3.7% of hyperparathyroidism cases [21], but hyperparathyroidism is mainly observed in benign parathyroid diseases [21–23].

At the best of our knowledge, only 11 cases of synchronous extrathyroidal parathyroid carcinomas and non-medullary well differentiated thyroid carcinoma are reported up to date in literature [24–34]. Indeed, our patient is the first documented case of synchronous non-medullary well differentiated thyroid carcinoma and parathyroid carcinoma with an intrathyroidal localization.

The rare constellation of synchronous thyroid carcinoma and intrathyroidal parathyroid carcinoma has prompted us to report this case and perform literature review to stress the challenging difficulties in diagnostic workup and surgical management.

## Case presentation

A 63 years-old Caucasian male patient, with a negative family history of thyroid diseases and without a personal anamnesis of neck irradiation, was referred to general surgery unit for multinodular goiter, bigger on the right side, complicated by a tracheal deviation and symptoms of compression.

Routine preoperative tests, unexpectedly, showed a mild serum hypercalcemia (13.3 mg / dL) [normal range (n.r.): 8.6–10.2)], hypophosphatemia [2.2 mg / dL (n.r. 2.7–4.5)], with no symptomatology of urolithiasis, myasthenia, osteoarthralgia, dyspepsia, constipation or psychotic depression. Serum parathyroid hormone (PTH) value was increased 159 pg / mL (n.r. 4.6–58.1), the urine calcium was 896 mg / 24 h (n.r. 100–400) and urine phosphorus was 1000 mg / 24 h (n.r. 400–1300). No kidney disorders were found thus a diagnosis of primary hyperparathyroidism was made.

**Fig. 1:**
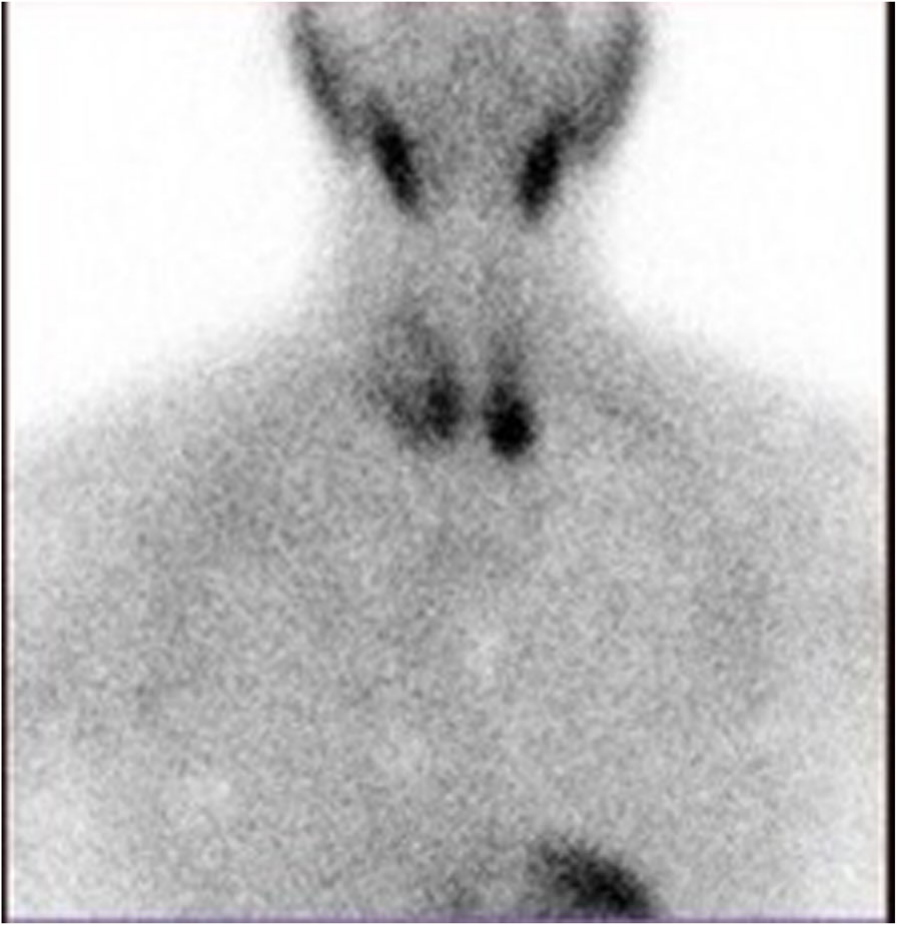
Scintigraphy with 99mtechnetium: Late hypercaptation at lower left pole of the thyroid, no mediastinal uptake

A neck ultrasound (US) exam confirmed a multinodular goiter without extra-thyroid lump: in particular, a 14 mm-diameter nodule with intravascular spot signal at Doppler exam was observed at the left posterior base of the thyroid. The parathyroid subtraction scintigraphy with 99mTechnetium and Sesta-Meta-iodo-Benzylguanidine showed a strong posterior signal at the left thyroid base in correspondence of the nodule (Fig. 1). No pathological signal was observed in other extrathyroidal sites.

Therefore, these observations suggested the suspect of an IP adenoma: the decision was made not to proceed with a needle aspiration, due to the risk of severe fibrosis that could complicate the final histological diagnosis [35].

Otherwise fine-needle cytology (FNC) on the major nodule of the thyroidal right lobe diagnosed Thy 2 according to Bethesda System.

The patient received an intravenous injection of 12.3 mCi of Tc99m-sestamibi approximately 1 hour prior to surgery. A total thyroidectomy with radio-guide technique using gamma probe after intraoperative sesta-MIBI administration and intraoperative PTH level evaluation was performed.

The intraoperative PTH level evaluation, performed with a chemiluminescence immunoassay, allows the determination of the serum bio-intact PTH value. The basal serum PTH level before thyroidectomy was 164 pg/mL. After 20 min from the surgery, the value decreased at 38 pg/mL and 30 min later at 19 pg/mL.

**Fig. 2:**
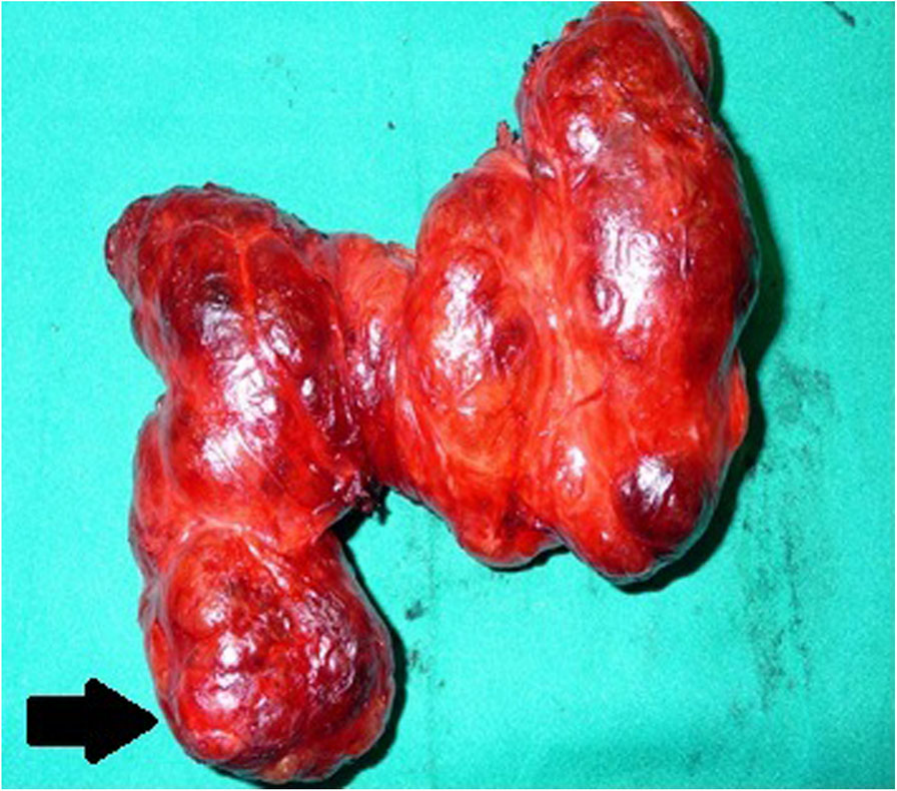
Posterior view of the operative specimen, node of the lower left pole of the thyroid subcapsular seat, reddish-yellow in color

The use of the gamma probe after surgery was essential to identify high radiation levels in the posterior left thyroid lobe, where a yellow-brown nodule was found (Fig. 2).

Conversely, the postoperative thyroid lodge did not show any radiation activity.

The patient was discharged on the third post-operative day without complications and with normal range serum values of PTH, calcemia and phosphoremia.

**Fig. 3:**
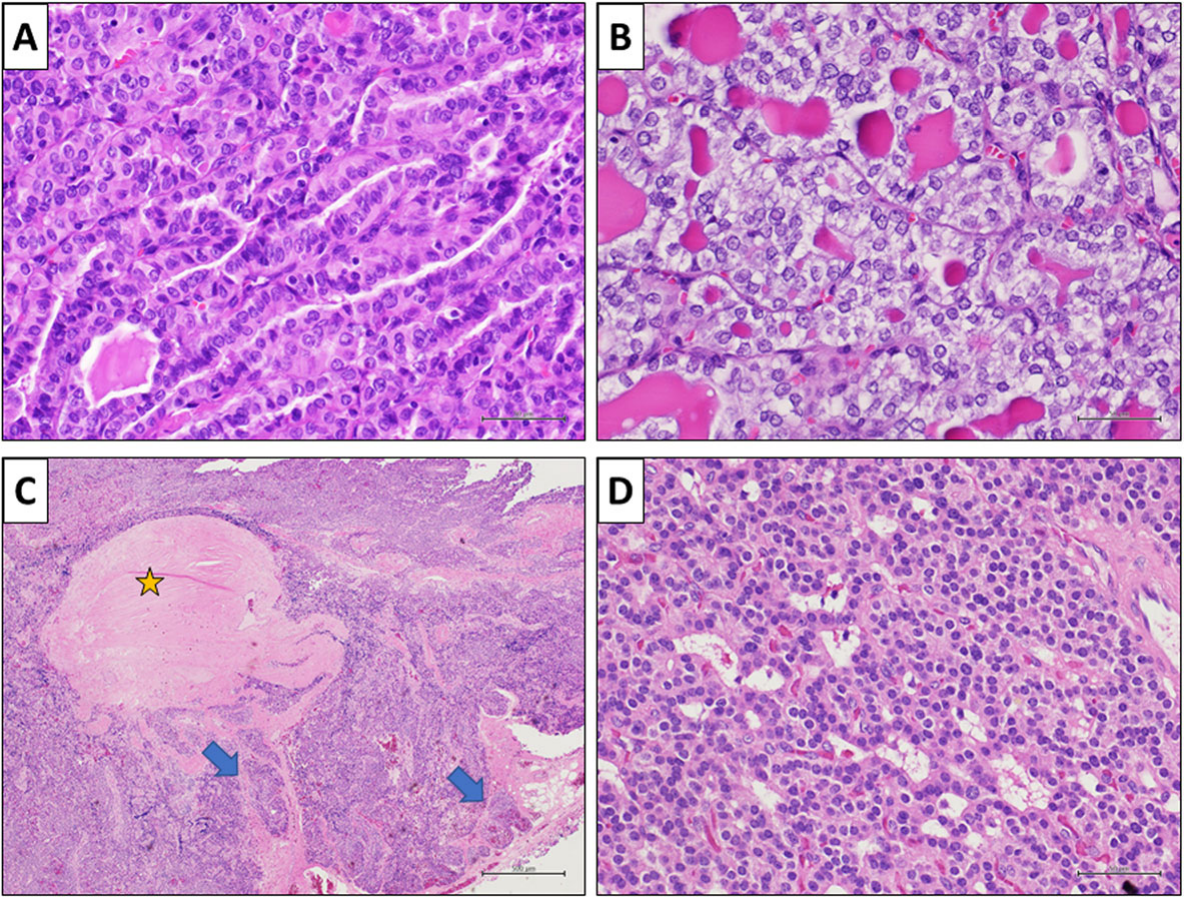
Histopathological finding. **a** Conventional papillary carcinoma. Histological evaluation showing a neoplasm with a papillary architectural pattern (H&E, original magnification 200x). **b** Follicular variant of a papillary carcinoma. Histological evaluation showing a neoplastic proliferation with a follicular architectural pattern and cytological atypia including ground-glass nuclei and nuclear grooves (H&E, original magnification 200x). **c**-**d** Parathyroid carcinoma. Histological evaluation showing neoplastic islets separated by thick bands of connective tissue (yellow star). The neoplasm shows an infiltrative growth in the adjacent soft tissue (blue arrows). Thyroid tissue is present in the lower right (C, H&E, original magnification 40x). The neoplasm is constituted by main cells (D, H&E, original magnification 200x). Abbreviation: H&E: hematoxylin and eosin

**Fig. 4:**
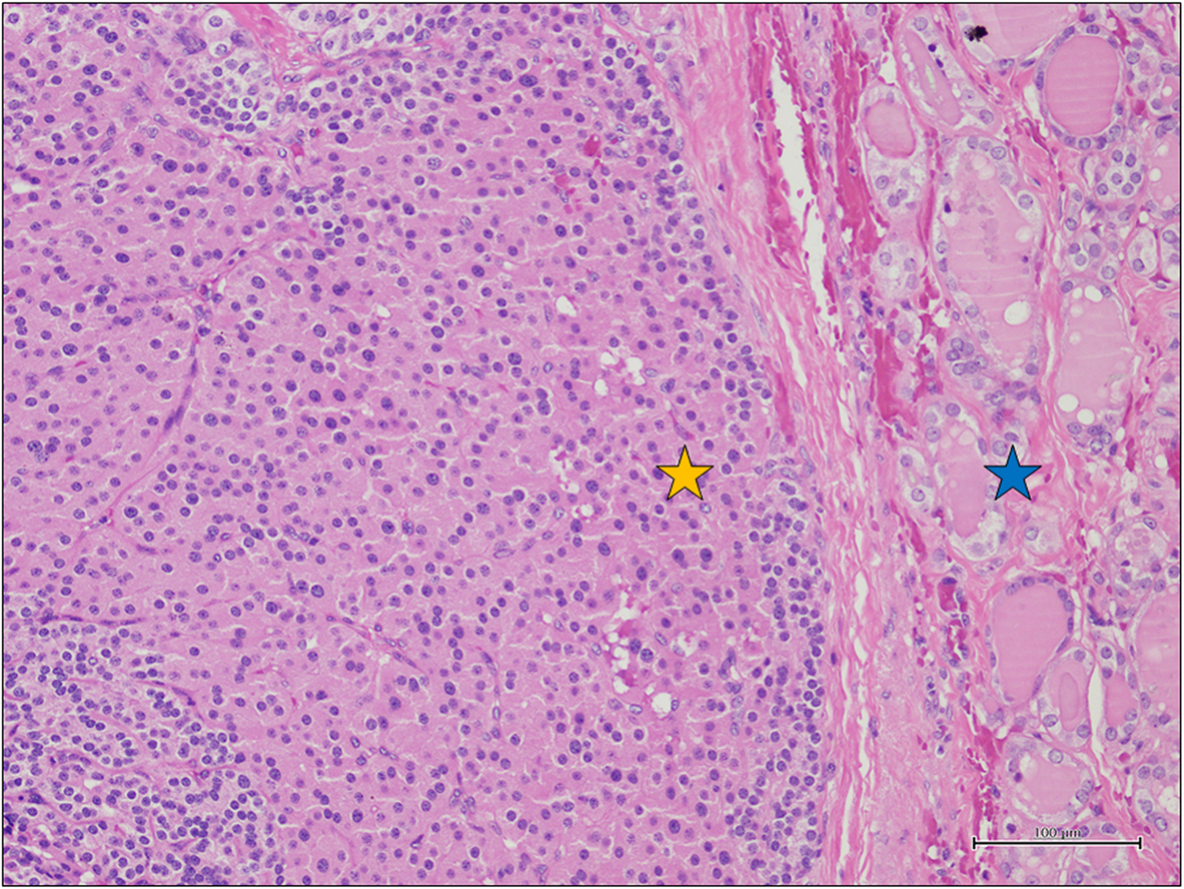
Nests of main cells (yellow star) associated with thyroid follicles (blue star) (H&E, original magnification 200x). Abbreviation: H&E: hematoxylin and eosin

Histopathological examination revealed a classic papillary carcinoma of 0.8 cm of diameter at the thyroidal left lobe (Fig. 3a), a follicular variant of a papillary carcinoma of 0.6 cm of diameter at the thyroidal right lobe (Fig. 3b) and a parathyroidal main cell carcinoma of 1.2 × 1.0 cm of diameter at the posterior thyroidal left basal lobe (Fig. 3c, d, Fig. 4). Photographs of histological slides were obtained using a digital acquisition system (Olympus DP2-SAL4).

Postoperatively, ^131^I ablation therapy was administered at a dose of 50 mCi.

The patient was followed up closely for both malignancies. US examination was performed every 6 months in the first year and every year later. TSH, FT3, FT4, Thyreoglobulin (Tg), Thyreoglobulin Antibodies, PTH, calcemia and phosphoremia was scheduled every 3 months.

Tg value was 6 ng/ml before I-131 therapy and remained < 0.2 ng/ml after treatment and for the 7 years follow-up; serum calcium, phosphorus and PTH levels remained in the respective normal ranges, with multiple negative neck US.

## Discussion and conclusions

In literature only 11 cases of simultaneous occurrence of extrathyroidal parathyroid carcinoma and thyroid tumor has been reported. To the best of our knowledge, the association of intrathyroidal parathyroid carcinoma and thyroid tumor has never been described.

We have illustrated this unique association and performed a review of the last 40 years’ literature on the association of synchronous parathyroid carcinomas and non-medullary well differentiated thyroid carcinomas.

Parathyroid carcinoma is usually sporadic or related to other diseases [(e.g., multiple Endocrine Neoplasia (MEN) 1 and MEN 2, single familial hyperparathyroidism (FIHP), hyperparathyroidism “jaw tumor” (HPTJT)].

Studies *of hereditary and syndromic forms of PC have revealed some genetic mechanisms underlying PC. Somatic mutations of CDC73* gene, involved in the Jaw tumor syndrome, would be the cause of hypercalcemic disorders and can be *identified in up to 70% of patients with PC. In one-third of cases the mutations are germline* [4, 36–38]*.*

Nearly 90% of parathyroid carcinomas are hyperfunctioning versus 7–10% of non-functioning forms [4]; this percentage is confirmed in our literature review of synchronous parathyroid carcinoma and well differentiated thyroid tumor with a value of 9.09%. In non-functioning forms the patient typically presents late because of “mass effect” of a palpable tumor or lymphadenopathies (15–30%), whereas the lung, liver or bone metastases are found in one third of the cases [3].

Instead, hyperfunctioning forms usually present with nephrolithiasis, myasthenia, psychotic depression, osteoarthralgia; 7–12% of the cases have a parathyroid crisis, usually with serum PTH value > 16 mg/dl, characterized by cardiac arrhythmias and altered consciousness that can lead to coma [4, 39].

Currently, clinical, biochemical or imaging criteria are not able to distinguish benignant from malignant diseases. A malignant diagnosis should be suspected in the following conditions: a) fast onset of acute symptoms; b) serum calcemia > 14 mg/dL, usually associated to parathyroid carcinomas in 65–75% of the cases; c) serum PTH level beyond 10 times the normal value (81% predictive positive value); d) metastasis detection on the radiological investigations [4, 39, 40].

The routine use of FNC is unnecessary and should be avoided because of fibrosis and related reactive changes that can make histologic interpretation of benign versus malignant disease difficult [35]. Moreover, the risk of lump rupture with malignant cell spread should be considered. Therefore, FNC is indicated only in case of recurrence suspicion.

In order to obtain a definitive diagnosis, histopathological examination is necessary using Shantz and Castleman criteria, such as trabecular pattern, mitotic figures detection, thick fibrous band and capsular and blood vessel invasion [41]. The immunohistochemical exam (PTH, GATA3, TTF-1, PAX8 and thyroglobulin) is useful to identify the uncertain forms [20].

According to our literature review summarized in Table 1, in 12 reports with synchronous parathyroid malignancy and thyroid disease, 11 patients were *hypercalcemic*, whereas the one reported by Savli et al. [28] was normocalcemic; in all of the 11 extrathyroidal cases reported there were no preoperative suspect of malignancy.

**Table 1:** Clinical features of 12 patients with synchronous parathyroid and thyroid carcinoma

Author	Sex	Age	Calcium (mg/dl)	PTh (pg/ml)	Parathyroid Size (cm)	Carcinoma location	Thyroid Carcinoma-	Surgical Treatment	Outcome
**Kurita** et al., 1979 [27]	F	68	12.2	6.3	4.2 × 3.2 × 2.4	Left	Papillary	En-bloc resection	Normocalcemia
**Christmas** et al., 1988 [29]	F	62	Hypercalcemia	Unknown	Unknown	Lower Unknown	Follicular	Unknown	Died from metastatic parathyroid carcinoma
**Savli** et al., 2001 [28]	F	47	Normal	ND	Unknown	Unknown	Papillary	Total thyroidectomy, parathyroidectomy (Excision of 2 hyperplastic glands)	Normocalcemia (1 year)
**Schoretsanitis** et al., 2002 [30]	F	55	14.2	> 1000	3 × 3	Left Lower	Papillary (Follicular variant)	En-bloc resection	Normocalcemia (2 years)
**Lin** et al., 2005 [24]	M	38	16.5	351	4x3x3	Left Lower	Papillary	Total thyroidectomy, left parathyroidectomy	Normocalcemia (6 years)
**Goldfarb** et al., 2009 [25]	M	58	14.4	2023	3.4 × 3.3 × 2.2	Left lower	Papillary	En-bloc resection	Normocalcemia after excision of contralateral parathyroid adenoma (1 year)
**Marcy** et al., 2009 [31]	F	42	12	180	Unknown	Right	Papillary	Total thyroidectomy, parathyroidectyomy	Normocalcemia
**Chaychi** et al., 2010 [26]	F	79	10.4	89	5	Left Lower	Papillary Multifocal	En-bloc Resection	Normocalcemia
**Zakerkish** et al., 2015 [32]	M	21	11.5	1311	Unknown	Unknown	Hürthle	Left thyroid lobo-istmectmy and cervical lymph node dissection	Persistent hypercalcemia after left lobectomy of thyroid and finally expire
**Dikmen** et al., 2017 [33]	M	57	11.4	184	21 × 11 and 30 × 20 retrosternal	Left inferior and anterior mediastinum	Micropapillary	Excission en-bloc with thoracoscopy, parathytoidectomy, left inferior and lobectomy ipsilateral	Normocalcemia
**Baek** et al., 2017 [34]	F	68	12.8	1247	40 × 30	Left inferior	Papillary (follicular variant)	Parathyroidectomy, left inferior and lobectomy left thyroid	Normocalcemia (6 months)
**Present case**	M	63	13.3	159	1.2 × 1	Left-intra-thyroidal	Bilateral: Papillary classic and Papillary variant Follicular	Total thyroidectomy and left inferior parathyroidectomy	Normocalcemia (7 years)

Preoperative diagnostic management is paramount to lead to the correct surgical approach of thyroidal and parathyroidal synchronous carcinomas.

In five out of 11 reported cases, a preoperative thyroidal FNC has been performed and was suggestive of malignancy; in the other six cases the diagnosis of malignancy was rendered at the histological examination.

In patients in whom the preoperative findings supported the diagnosis of thyroidal carcinoma, the planned surgical technique was to perform an en-bloc resection of parathyroids with total thyroidectomy, while ipsilateral and/or contralateral hemithyroidectomy was reserved to patients with the suspect of malignancy or with follicular thyroid neoplasm (Table 1).

Our experience and literature review assess that nowadays the treatment of this synchronous oncologic disease is incredibly challenging and specific guidelines are still missing.

In our case the preoperative clinical findings supported the diagnosis of a multinodular goiter (FNC on the major nodule Thy 2 according to Bethesda System), complicated by a tracheal deviation and symptoms of compression with a suspect of a parathyroidal adenoma.

Therefore, our patient underwent a total thyroidectomy because of his diagnosis and according to the *gold standard surgical procedure*. Moreover, this approach allowed the incidental discovery of the multicentric thyroid malignancy and the accidental excision of intracapsular parathyroid carcinoma.

In our experience, radioguided technique (gamma-probe) and the intraoperative PTH dosage turned out indispensable, in order to assess the completeness of the excision.

For the parathyroid carcinoma, indeed, bloc excision of the mass and any involved surrounding structures, preserving the integrity of the capsule, is the only curative approach for long term survival.

Ipsilateral lymphadenectomy of the central and/or laterocervical compartment is recommended in case of lymph node involvement evidence [3].

Recurrence of parathyroid cancer occurs in a range between 49 and 60% of the cases, with a mean time of 2.5–4.8 years after surgery [3]. Negative prognostic factors include young age and lymphadenopathies or metastases present at the onset. The most common recurrences are locoregional or distant metastasis involving lung, liver and bones [3, 39, 40, 42, 43].

Because of the exceptional rare condition for parathyroid cancer, there is no general consensus on treatment and follow-up [44]. According to the literature, patients should be followed up life-long measuring calcium and PTH and performing regular US surveillance [38, 44]. Moreover, our treatment strategy was based also on the evidence of thyroid malignancy; even if histopathological examinations revealed a multifocal thyroid microcarcinoma without signs of aggressive histology, the specific features of the patient, such as age > 60, sex, Tg value and patient preference have prompted us to recommend RAI remnant ablation, also according to nuclear counselling.

At 7 years-follow up, there is no evidence of both thyroidal and parathyroidal malignant recurrences or parathyroid hyperfunction.

According to our experience and recent studies, radical surgical approach is currently the goal standard of treatment for recurrences [3, 39, 40, 42, 45–47].

In fact, according to literature, only a few authors [48–54] attest the usefulness of radiotherapy and chemotherapy in improving rate of recurrence and survivor but the efficacy of adjuvant therapies has recently been disappointed in the management of recurrent parathyroid carcinoma, even if multicentric studies for improved long-term outcomes are still lacking [46, 47].

Nevertheless it is paramount to consider that the causes of death in patient with lymphatic recurrence or secondary malignance are more closely related to chronic and refractory hypercalcemia than the presence of metastatic mass [54].

In conclusion, our experience emphasizes that in case of intraoperative suspect of parathyroid carcinoma, an en-bloc resection of the parathyroid tumor and the ipsilateral thyroid lobe is mandatory, even if there is no evidence of thyroidal involvement.

This approach avoids a successive neck surgical procedure that could be justified by the high local and vascular aggressiveness of the carcinoma and could be related to serious and frequent complications as recurrent laryngeal nerve injury [3, 4, 45].

## Data Availability

The datasets used and/or analysed during the current study are available from the corresponding author on reasonable request.

## Abbreviations

IP: Intradthyroidal parathyroids
FNC: Fine-needle cytology
n.r: Normal range
US: Ultrasound
Tg: Thyreoglobulin
PTH: Parathyroid hormone
PC: Parathyroid carcinoma
MEN: Multiple endocrine Neoplasia
FIHP: Single familial hyperparathyroidism
HPTJT: Hyperparathyroidism “jaw tumor”
